# Scientific writing is not a murder mystery

**DOI:** 10.7554/eLife.112853

**Published:** 2026-08-19

**Authors:** Charlotte A Dodson

**Affiliations:** 1 Department of Life Sciences, University of Bath Bath United Kingdom

**Keywords:** murder mystery, science writing, publication, academia, literature, sherlock holmes, None

## Abstract

What murder mysteries can tell us about how not to write a scientific article.

It’s project time again. Once more I find myself in my office with a slightly embarrassed and politely amused research student giving the same feedback as last year: ‘You’re not writing a murder mystery.’

Each year, thousands of students across the world create their first piece of scientific writing, often the first draft of a research paper or a dissertation reporting the results of a short project. I cannot believe my students are alone in struggling – at least at first.

There is no shortage of advice, blog posts and books available on scientific writing. Some of these discuss the writing process itself (how to get words on the page), some give enormous detail on structure. Here, I take a different angle and consider the difference between the structure of scientific writing and another form of writing with which most – if not all – readers will be familiar: the murder mystery.

In a murder mystery, the writer is creating tension and suspense. We are drawn into a different world by the use of scenes and dialogue, not description. We are *shown* what is happening, not *told* about it. And the golden rule – the breaking of which has led to a new genre of reverse or inverted detective fiction – is that we never find out the key result of who perpetrated the crime until just before the end.

I have found the similarity between many student first drafts (or even submitted projects) and a murder mystery novel a source of both amusement and exasperation. In many cases, I find the key result of the research revealed somewhere towards the end. This plot twist can cause me to re-evaluate everything that I have read up until that point. Sometimes I am cast in the role of the naïve observer, coming along for the ride in every turn of an investigation without a clear sense of what the hero expects to learn from their current action. Occasionally I am asked to share the heartache of every failed assay and repeatedly encouraged to hope that another attempt may result in a different outcome.

Contrasting with this, a scientific paper is closer to the inverted detective novel. We summarise the scenario, the crime and the actions of the detective: first in the abstract and again at the end of the introduction. We declare the criminal – and the repercussions of this case – up front. The majority of our writing provides detailed evidence – with or without subsidiary plot lines – to support our conclusion. At the end, we remind everyone what we have done once more.

Unlike a mystery novel, in which language is deliberately chosen to create suspense, the language of a paper is deliberately chosen to be neutral and to prioritise clarity. Plot lines – and any key information withheld between chapters – are carefully untangled and collated. Material is grouped thematically, not presented as a diary. Control experiments – even if carried out late in the day – are presented alongside the result. All showmanship is removed and is replaced by logical and/or technical elegance. Any tours de force comes in the form of thorough experimental data.

In a scientific paper, there are no naïve narrators who conceal the thoughts and actions of other characters. Instead, the logic of the brilliant detective is exposed step by step alongside their observations. The rationale for every question asked is made clear by the use of stylistic constructions such as Purpose – Action – Result – Conclusion: ‘*In order to … we carried out … This showed … (figure X), meaning that…*’.

In order to illustrate these differences in a light-hearted fashion, I have rewritten Sherlock Holmes’ adventures in *The Hound of the Baskervilles* (Conan Doyle, 1902) in the style of a scientific paper (Supplementary file 1). I have provided a second, colour-coded file which makes the different stylistic statements explicit (Supplementary file 2).

The resulting paper is divided into the five traditional sections that I use in my own work (abstract, introduction, results and discussion, conclusions and methods). The abstract follows the format of background, knowledge gap, results (including methodology), headline conclusion and implications for the field (Nature, 2019). Instead of a full literature review, the introduction selects the information necessary to understand the context and content of the work that follows. Much of this is not found in the original novel. The introduction ends with a summary of the work carried out, the main conclusions and a statement of significance.

The results and discussion of the paper present the majority of the original novel but differ considerably in style. Key differences include a thematic ordering of material, the use of sub-headings, and a deliberate ignorance of differences in knowledge between characters. Within each sub-section, I have consciously used the Purpose – Action – Result – Conclusion format. Information is presented diagrammatically where possible. The conclusions section summarises the work carried out, the main take-home points, and relates this back to the context given in the introduction. Methods information is made explicit.

In contrast with the original novel, the vocabulary in this paper has been chosen to convey factual information in a neutral tone. Wherever possible, the text uses the active voice, relatively short sentences and commonly used words (Doumont et al., 2014; Nature, 2026; Robens, 2022; Turbek et al., 2016). Some material is expanded or given increased prominence compared with the original text, while much is condensed. Unnecessary details are removed.

The resulting work is around 10% of the length of the original novel. It lacks mystery, suspense and surprise. It considers *why* the reader may wish to read the full paper (i.e., reading is assumed to be for a purpose, not pleasure) and contains nearly 650 words of material which is irrelevant to, presumed, or conveyed implicitly in the text of the original novel.

Dr Watson will shudder at my prosaic form. Arthur Conan Doyle may or may not be turning in his grave. Nevertheless, I rather hope that Sherlock Holmes, that intense and confident proponent of ‘the scientific method’, will appreciate the joke. Holmes is noted for dismissing offhandedly the ‘romanticism’, which Watson included in his accounts of their joint adventures (Conan Doyle, 1890). He much preferred to focus on solving the problem before him using interrogation, observation and logic.

The purpose of much scientific writing is to advocate a position and advance knowledge by communicating context, conclusion and evidence against shared standards. It is to be Holmes (albeit with less arrogance) rather than Watson. I hear Watson’s story – equally valid, but with a different purpose – in the lab, in handwringing over coffee, and in conversations in my office. I thoroughly enjoy it in popular scientific biography. The Holmes monograph is closer to what I read in scientific journals. Although both narratives recount the solution to an outstanding problem, every mystery can be demystified when the answer is known. Even *The Hound of the Baskervilles* can be written in the style of a scientific paper – however absurd this may initially appear.

## Additional files

Supplementary file 1. *The Hound of the Baskervilles* rewritten in the style of a scientific paper.

Supplementary file 2. Copy of Supplementary file 1, colour-coded to make the different stylistic statements explicit.

Supplementary file 3. List of seventy-five perfumes considered by Sherlock Holmes.
