# Supplementary material for: Scientific writing is not a murder mystery
Source: eLife. 2026 Aug 19;15:e112853. doi: 10.7554/eLife.112853 (PMC13489623; doi:10.7554/eLife.112853)
Supplement: Supplementary file 2 [file elife-112853-supp2.pdf]

ARTICLE

Superstition within the Baskerville family is a poor predictor of murderer identity

Received 13th July 2026,
Accepted 17th July 2026

DOI: 10.7554/eLife.112853

Charlotte A Dodson [a]

In literature, as in real life, many bereaved families seek answers following a violent death. In 19th century English Society, one solution was to seek the services of the fictional detective Sherlock Holmes. Following the unexpected death of Sir Charles Baskerville, Mr Holmes used interview, observation, desk-based enquiry and advanced logical deduction to determine that 'Jack Stapleton' was a pseudonym for Mr Jack Baskerville, heir apparent to the Baskerville baronetcy. A sting operation provided direct evidence of attempted murder on the part of Mr Stapleton. Fictional deaths in literature need not rely on the supernatural for interest and can achieve success using human agents, the scientific method, and conventional motives including greed. This recipe may inspire subsequent literary output.

Introduction

In the 19th century, English language popular literature reflected many aspects of contemporaneous Western societal interest. Most relevant here are the genres of social or domestic realism [1-6], a fascination with wild remoteness and the supernatural [6-10] (even if ultimately resolved rationally [7]), and scientific advance [9-12] (Figure 1). In some works the scientific advances were either exaggerated or sensationalised for literary dramatic effect [9,10,12]. Nevertheless, the contextual setting of science often reflected the rationalist or empiricist explanations of natural phenomena (or technological applications of these) which were becoming an increasing part of societal discussion [2,4,13].

By the late 1880s, one emerging implementation of both rationalist and empiricist thinking in literature was the investigation and resolution of crime (Figure 1, red text). Principle characters in these works included both professional investigators [14-16] and gentleman amateurs [17,18]. Irrespective of the nature of the investigator, these works were firmly set within the genres of realism and / or scientific advance without reference to either romantic gothic scenarios or to the supernatural.

Arthur Conan Doyle's *The Hound of the Baskervilles* [19] differs in this respect. Sherlock Holmes has previously been presented as an eccentric hero of both considerable physical prowess and intellectual ability [20,21]. Holmes' characteristic traits include an eye for detail, self-belief bordering on the arrogant, considerable monotropism, and enthusiasm for his area of technical expertise (which he summarises as 'the scientific method'). Professionally, Mr Holmes is a self-styled consulting detective with documented success in solving mysteries on behalf of multiple clients, providing faster and more accurate outcomes than those of the professional police service. This has led to Mr Holmes being seen by victims and their families in 19th Century London and beyond as an individual of choice to approach for help when solving a crime or resolving a mystery. Mr Holmes shares his London lodgings with Dr John Watson, a medical practitioner of a similar age.

The events in this Adventure primarily take place on or near to Dartmoor, a region of approximately 370 square miles (950 km^2) in Devon, UK with a reputation of bleak wilderness [22,23]. The moor itself is grazed and is also a historical landscape containing (among others) Bronze Age hut circles and evidence of tin mining from prehistory onwards. The landscape giving rise to the reputation exploited here includes granite tors, grass moor and heathland, upland peat bogs and valley mires (waterlogged deep peat found in steep-sided valleys, natural basins and abandoned tin workings). HMP Princeton, a men's prison originally built to house French (Napoleonic) prisoners of war, opened in 1809 and is situated within Dartmoor [24]. During the late Victorian era Princeton was notorious for housing violent civilian offenders.

Colour key

Grey	Background
Teal	Knowledge gap
Red	Purpose
Cyan	Results
Yellow	Methods (abstract) / summary of work carried out (introduction & conclusion) / action (results & discussion)
Green	Conclusions
Magenta	Implications for the field (abstract) / statement of significance (introduction) / relating back to context in introduction (conclusion)

[a] Department of Life Sciences, University of Bath, Claverton Down, Bath BA2 7AY, UK. c.a.dodson@bath.ac.uk

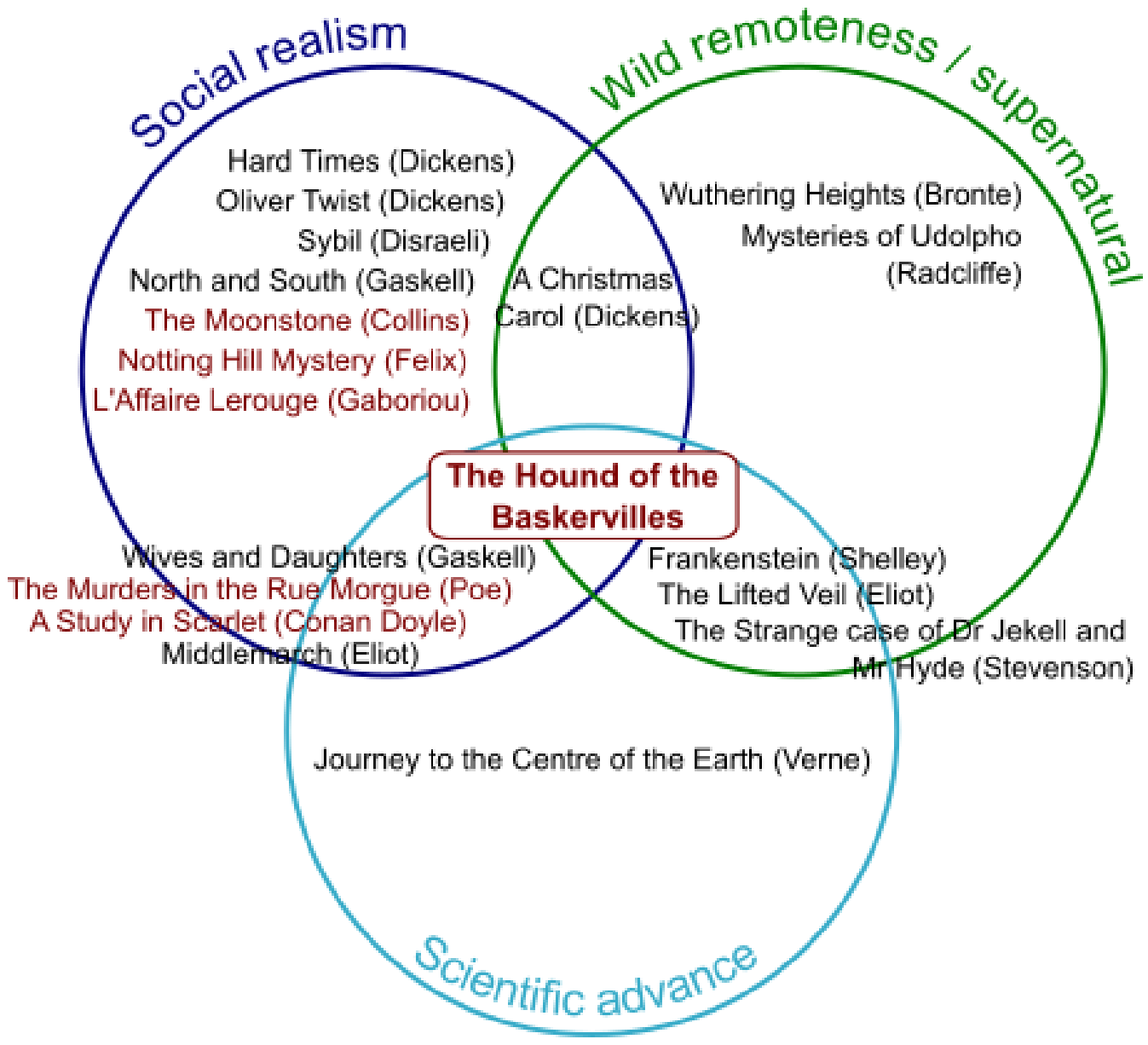

Figure 1: Three genres of 19th Century fiction with example texts published prior to 1889 (the setting of The Hound of the Baskervilles). Red text indicates works of detective fiction. NB Although The Mysteries of Udolpho was published just prior to the 19th century (in 1794), it is included here due to its influence on later works.

The family central to this Adventure, the Baskerville family, is a fictional hereditary baronetcy with a family seat (Baskerville Hall) on the edge of Dartmoor. The legendary poor behaviour and death of an earlier occupant of the Hall, Hugo Baskerville, during the late 1640s coupled with the unexpected violent deaths of subsequent baronets gave rise to the belief that the family was haunted by a supernatural hound [25]. This superstition – known both within the family and in the surrounding geographical neighbourhood – was reinforced shortly before this Adventure by independent verbal reports from three working men. Each account details the sighting of a gigantic hound with flaming jaws on Dartmoor. Sounds consistent with such a creature were reported more widely.

Very recently, the late Sir Charles Baskerville, a retired mining millionaire with a history of both local philanthropy and cardiac disease, was found dead face-down in the grounds of his house. An autopsy gave the cause of death as cardiac arrest, although the local press reported human cries prior to discovery of the body [26]. Consistent with some disturbance, Dr James Mortimer, a medical practitioner from the nearby fictional hamlet of Grimpen, testified at the inquest that the late Sir Charles' facial expression was distorted to the point of unrecognizability [26]. Dr Mortimer is also an executor of the estate of the late Sir Charles.

In this Adventure, Mr Holmes applies his talent for observation and logical deduction to events within the Baskerville family. Dr Mortimer is concerned that the best explanation of the late Sir Charles' death is supernatural. He engages Mr Holmes on behalf of Sir Henry Baskerville, the new baronet, recently arrived in London from Canada, to advise on his best course of action. After interviewing both Sir Henry and Dr Mortimer, Mr Holmes contrasts the loss and subsequent return of a new boot of Sir Henry's in London with the sustained loss of an old one. He uses this information to reason that a mortal – not satanic – dog is likely to be involved. Mr Holmes further analyses the scent of a warning note received by Sir Henry to suspect educated women within Sir Charles' social circle – and / or their wider households – of making a human contribution. Using wider desk-based enquiries and the information contained in subsequent written reports sent from Baskerville Hall by Dr Watson, Mr Holmes determines that Mr Jack Stapleton is a pseudonym for Mr Jack Baskerville, heir apparent to the Baskerville baronetcy. Mr Holmes' own covert observations uncover an intimate relationship between Mr Stapleton and Mrs Laura Lyons, the estranged daughter of a local eccentric. A subsequent sting operation further supports the conclusion that Mr Stapleton is responsible for the death of the late Sir Charles Baskerville, the death of an escaped convict on Dartmoor and an attempt on the life of Sir Henry Baskerville.

This Adventure contrasts with previous examples of Western detective fiction by sustaining a potentially supernatural scenario within a realistic and rationalist / empiricist framework. In doing so, it asserts the greater explanatory power of the rational over the supernatural. We expect that it will set the tone for success in multiple future literary endeavours.

Results and Discussion

The late Sir Charles was preoccupied by the Baskerville family legend and planned to depart Dartmoor for London

In order to gain relevant contextual information about the case, Holmes first interviewed Dr Mortimer in his apartment at 221B Baker St. In accordance with current best practice, Mr Holmes started the interview with open questions [27]. Dr Mortimer reported that – three weeks before the late Sir Charles' death – Dr Mortimer had personally witnessed a reaction of horror on Sir Charles' part to what Dr Mortimer presumed to be a large black calf passing the head of the drive at Baskerville Hall. Based on concerns for Sir Charles' mental health, specifically arising in the context of Sir Charles' reaction to the incident and the connection made between this and the family legend, Dr Mortimer recommended that Sir Charles leave Dartmoor to spend a few months in London. This course of action was supported by Mr Stapleton, a local naturalist and one of only two other educated gentlemen in the area.

The late Sir Charles lingered outdoors on the night of his death

A mixture of active listening and closed questions carefully constructed to elicit specific information led Holmes to discover that the late Sir Charles had been present at a wicket-gate leading from the grounds of Baskerville Hall onto Dartmoor on the night of his death (the night before his planned departure for London; Figure 2A). Although it appeared out of character (Sir Charles was known to avoid Dartmoor after dark), on this occasion he had remained at the gate long enough for the ash to drop twice from his cigar. This was estimated by both Holmes

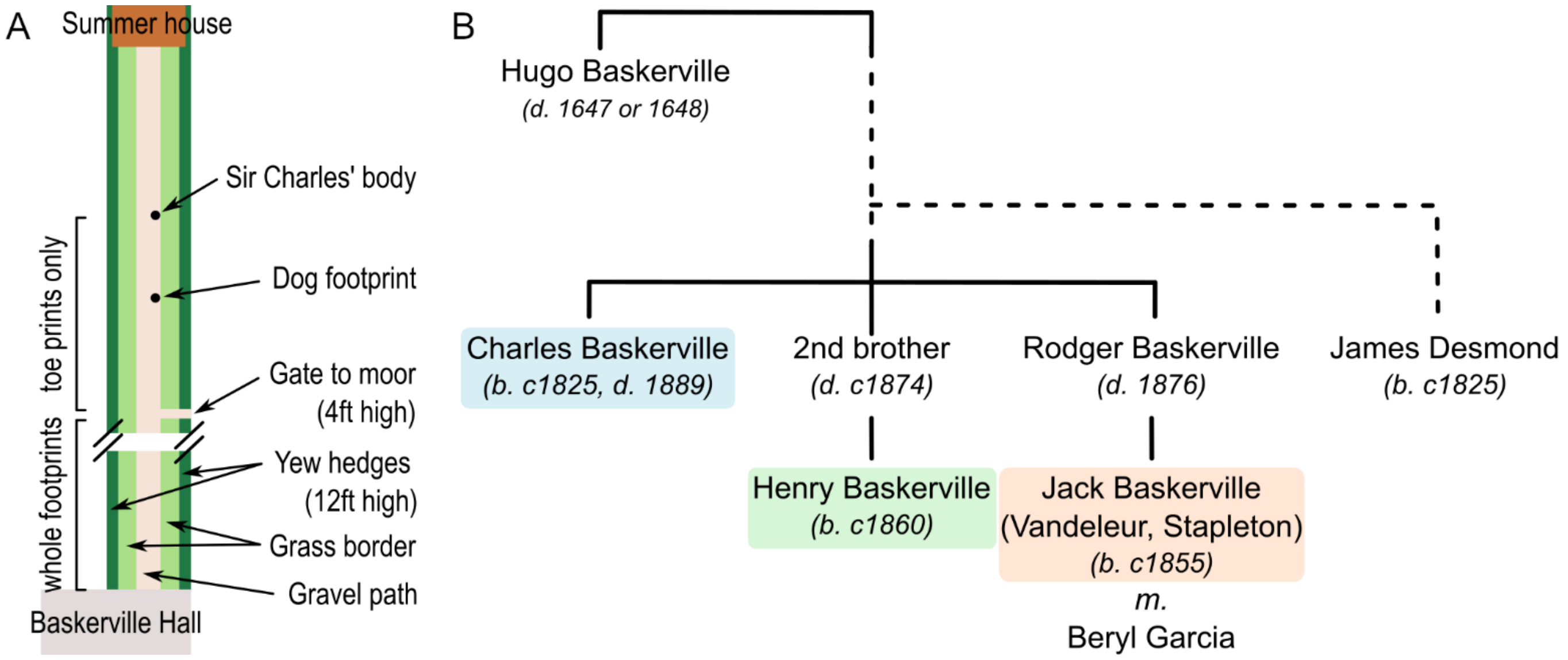

Figure 2: Contextual information on Baskerville Hall and family. (A) Diagram of the Yew alley at Baskerville Hall. Characteristics of the late Sir Charles' footprints are indicated on the left. (B) Baskerville family tree highlighting relatives of Hugo Baskerville relevant to this Adventure. The late Sir Charles is highlighted in blue, his nearest known relative (his nephew, Sir Henry Baskerville) in green, and his second nephew (Jack Baskerville, also known as Stapleton) in orange. The Rev. James Desmond – a distant cousin of the late Sir Charles – is heir to the baronetcy after Jack Baskerville.

and Mortimer to be around 5-10 mins.

A large dog, of likely earthly nature, was present in the vicinity of Sir Charles on the night he died and may have frighted Sir Charles

Without specific prompting, Dr Mortimer reported that at approximately 1 am on the night of Sir Charles' death, he found a footprint on the gravel path around 20 yards from Sir Charles' body (Figure 2A). He described the appearance of this print as that of a gigantic hound. There was no evidence that the dog had approached or interfered with the body, and Holmes reasoned that – since a hound will not approach a dead animal – Sir Charles was dead when the footprint was made.

At the inquest into the late Sir Charles' death, John Barrymore, butler at Baskerville Hall and the person who discovered Sir Charles' body, reported that Sir Charles' footprints had changed from regular full prints between the Hall and the moor gate to those of his toes alone between the gate and the summer house (Figure 2A) [26]. Mr Barrymore inferred that Sir Charles had been walking on tiptoe, although Holmes preferred the explanation that Sir Charles had instead been running. Consistent with the distorted face reported by Dr Mortimer [26], Holmes hypothesised that Sir Charles had observed something from the gate which terrified him to the extent that he ran away from the Hall without logical thought. Sir Charles' known preoccupation with the family legend and the observation of a large canine footprint that same night together provided a possible causal explanation.

In an additional interview with Holmes, Sir Henry Baskerville, the new baronet, responded to an open question about 'anything out of the ordinary routine of life' with the information that he had lost two single boots at his London hotel within 24 hrs of each other. One boot was unworn, the other older. The new boot was lost first and was returned within 48 hrs, the old boot was not retrieved. This observation led Holmes to hypothesise that the age of the missing boot was important. Based on the premise that a dog will track or otherwise search out an individual only if provided with an example of the scent to follow, Holmes concluded that any dog involved in the current Adventure was likely to be earthly in nature (as opposed to the putative supernatural hound of the Baskerville legend).

The late Sir Charles had a substantial estate and few close surviving relations

In order to determine the magnitude of any financial motive for Sir Charles' death, Holmes used closed questions to establish the value of Sir Henry's inheritance. Sir Henry confirmed this to be £740,000 (equivalent purchasing power to £83m (approx. $112m) in 2026 [28]), a total of which he was unaware until his arrival in London.

Holmes built upon this with further closed questions to determine the identity of any beneficiaries or potential beneficiaries of Sir Charles' estate. Neither Dr Mortimer nor Sir Henry was aware of any close living relation other than Sir Henry. Sir Henry's father had died young, and Rodger Baskerville, the youngest brother of the three, was believed to have died unmarried and without issue over a decade earlier. The next in line to the hereditary title was therefore a distant cousin of the late Sir Charles, the Rev. James Desmond (Figure 2B). Holmes discounted any financial motive on the part of the Rev. Desmond since the Rev. Desmond had refused an annual income from Sir Charles on at least one occasion and was described by independent witnesses as being of an amiable disposition. Desmond's age, occupation and modest lifestyle were likely also taken as evidence of good character, in accordance with societal norms.

Around £260,000 from the late Sir Charles' estate had been left to individuals and charities. The most substantial bequests were to Dr Mortimer (£1,000, equivalent to £112,000 ($150,000) in 2026 [28]) and to Mr and Mrs Barrymore, butler and housekeeper to the late Sir Charles (£500 each, equivalent to £56,000 ($75,000) [28]). Although all three individuals had financial motives for Sir Charles' death, this was mitigated by engaging Holmes (Dr Mortimer) and by societal expectations following four generations of service to the Baskerville family (Mr & Mrs Barrymore).

An unknown person or persons, likely including a lady, observed Sir Henry Baskerville in London

At their first meeting, fewer than 24 hrs after arriving in London, Sir Henry asked Holmes' advice about a warning note that he had received earlier that day. The letter consisted of typewritten words stuck onto paper. In order to deduce further information about the sender of the letter, Holmes observed the typeface, addressing and scent of the note.

By matching the font and text with that used in the *Times* newspaper leader column the preceding day, by considering the likely socio-economic profile of readers of this newspaper, and by observing the poor state of the ink bottle and pen used to write the address, Holmes reasoned that the note had been created by an educated person using hotel facilities. The presence of a faint scent of white jessamine indicated that the sender was female. A list of the 75 perfumes considered in this identification is provided in Supplementary file 3.

Based on this this information, Holmes highlighted two women in Sir Charles' social circle as potentially involved: Miss Beryl Stapleton, sister of the naturalist referred to above, and Mrs Mortimer, wife of Dr Mortimer. It is unclear at what point Holmes also considered the potential involvement of Mrs Laura Lyons, the estranged daughter of Mr Frankland, a local eccentric and amateur astronomer.

Consistent with a written warning to Sir Henry in London by a lady, Dr Watson later received a verbal warning in person about his presence in Devon from Miss Stapleton. It transpired that at the time of the warning Miss Stapleton had mistaken Dr Watson for Sir Henry Baskerville although it is unclear what, if any, significance Holmes attributed to this.

Mr Jack Baskerville, nephew to the late Sir Charles, and his wife were living local to Baskerville Hall under an assumed name

Criminal motivation may be hidden to the casual observer, particularly if this is what the perpetrator intends. In order to uncover hidden motivation in this case, Holmes carried out a panel of routine background checks on several residents local to Baskerville Hall and on the deceased siblings of the late Sir Charles. Dr Watson reported that Mr Stapleton had previously owned a school in the north of England and Holmes used this information to identify Stapleton and his sister as Mr and Mrs Vandeleur, former proprietors of St Oliver's school in east Yorkshire. The school had been established following the arrival of the Vandeleurs in the UK from Central America and, like Mr Stapleton, Mr Vandeleur had considerable expertise in lepidoptery (entry for *Papilio vandeleuri* in ref [29]). A photograph of Mr and Mrs Vandeleur taken in York in 1884 confirmed that they were the same individuals as Mr and Miss Stapleton.

A true sibling relationship between Mr and Miss Stapleton is supported by Mr Stapleton's offer of marriage to Mrs Laura Lyons upon her future divorce (see below). However, a marital relationship is supported by the absence of shared physical characteristics within the couple (Mr Stapleton is described by Dr Watson as short with a fair complexion and hair and grey eyes while Miss Stapleton is described as tall with dark complexion, hair and eyes) and by Dr Watson's observation that Mr Stapleton was highly possessive of his apparent sister. One manifestation of this possessiveness was his prohibition of any immediate relationship between her and Sir Henry.

Holmes further matched information on Mr Vandeleur with information he had received about the late Sir Charles' youngest brother, Mr Rodger Baskerville. Contrary to the belief of the late Sir Charles, Holmes uncovered evidence that Rodger Baskerville had married in South or Central America and was survived by his son (Figure 2B, orange box). Ancillary records established that Mr Jack Stapleton was a pseudonym for Mr Jack Baskerville, nephew to the late Sir Charles, and a fugitive from Costa Rica following the misappropriation of public money. The identity of Mr Stapleton as Mr Jack Baskerville is further supported by a strong facial resemblance between Mr Stapleton and a portrait of Hugo Baskerville, a resemblance previously reported for Mr Rodger Baskerville. This identification, together with evidence of financial insecurity on the part of the Stapletons, provided a considerable motive for ill-will towards both Sir Henry and the late Sir Charles Baskerville.

Mrs Barrymore was aiding her brother, a fugitive convict on Dartmoor

From shortly after their arrival by train in Devon, Sir Henry and Dr Watson were aware of increased security in the form of soldiers and prison warders searching for a murderer escaped from Princeton. Both this and evidence of considerable concealed sorrow on the part of Mrs Barrymore provided a source of noise in the observations of Dr Watson until he and Sir Henry witnessed Mr Barrymore signalling with a candle to an unknown person on the moor. Under duress, Mrs Barrymore identified the person on the moor as her younger brother, the missing convict, whom she and her husband had been providing with food. They had also passed on cast-off clothing which Sir Henry had gifted to Mr Barrymore.

This discovery explained many of the anomalies which Dr Watson had observed about Baskerville Hall and effectively eliminated the Barrymores from the investigation. Mr and Mrs Barrymore assured Sir Henry and Dr Watson that Mrs

Barrymore's brother would leave the country within a few days without further criminal activity.

The fugitive convict, dressed in Sir Henry's clothes, fell to his death in a state of considerable fear

On the night of discovering the convict's identity, both Dr Watson and Sir Henry observed the silhouette of a second – unknown – individual on the moor against the skyline. The presence of a covert gentleman resident within the ruined huts on Dartmoor was later confirmed by reports from the convict recounted to Dr Watson by Mr Barrymore, by Mr Frankland's report of regular visits to the moor by a supporting child, and by Dr Watson's direct observation of the child agent using Mr Frankland's telescope.

Dr Watson found the covert presence of any individual on the moor suspicious and was further concerned that this unknown resident might bear ill-will towards Sir Henry. As soon as Mr Frankland's observations indicated the correct area to search, Dr Watson therefore set out to find and interview the individual concerned. This resulted in him discovering Holmes' covert presence and camping place.

While conversing at Holmes' moorland hut shortly after dusk, both Holmes and Dr Watson heard the screams of a man. The screams moved rapidly in their direction and were accompanied by the sound of a baying dog. Both men moved to investigate further.

Following the sudden end to the screams, Holmes and Watson discovered the body of the fugitive convict face down at the base of a cliff, dressed in clothing originally belonging to Sir Henry Baskerville. The convict had sustained head injuries consistent with a considerable fall. Consistent with the suspicions of Holmes, Mr Stapleton met Holmes and Dr Watson by apparent chance close to the convict's body. Mr Stapleton initially expressed concern for Sir Henry, whom he had invited to visit that evening, although upon close observation he identified the convict as an unknown man. The combination of an apparently coincidental meeting in the dark, expression of concern for Sir Henry, and the death of a man wearing Sir Henry's clothes, together gave weight to Holmes' hypothesis that Mr Stapleton was planning to engineer Sir Henry's death.

Mr Stapleton promised marriage to Mrs Laura Lyons and induced her to make an appointment with the late Sir Charles on the night of his death

In order to thank Dr Watson and Sir Henry Baskerville for remaining silent about the convict, and prior to the convict's death, Mr Barrymore volunteered information about the night of Sir Charles' death that had only recently come to light. Shortly before Sir Henry's arrival in Dartmoor, Mrs Barrymore had discovered the postscript of a letter in the fireplace of the late Sir Charles' study. This arranged a meeting between the writer and Sir Charles on the night of his death. This meeting was arranged at the gate between Baskerville Hall and the moor (Figure 2A) for 10 pm. The postscript was signed 'LL' and Mr Barrymore additionally recalled that – unusually – Sir Charles had only received a single letter the morning before he died. The letter had been addressed in a woman's handwriting and had been postmarked Coombe Tracey (the nearest town to Baskerville Hall).

Dr Watson found the time and location of the proposed meeting suspicious and therefore asked Dr Mortimer – who was in professional contact with a wide range of individuals – for help in identifying individuals with the correct initials. As a result of this, Dr Watson interviewed Mrs Laura Lyons who, coincidentally, lived in Coombe Tracey. Mrs Lyons was separated from her husband and had set herself up in business with the support of a small number of local philanthropic gentlemen. Her supporters included both Mr Stapleton and the late Sir Charles.

Mrs Lyons explained that she was seeking to divorce her husband. She had written to Sir Charles with some urgency on the day before his departure for London seeking Sir Charles' potential assistance with the associated legal fees. Mrs Lyons had discounted a meeting the following day due to Sir Charles' likely travel arrangements and had considered a late-night informal meeting at the moor gate more socially acceptable than a formal visit to a bachelor at a similar hour. She stated that she had neither attended nor cancelled the meeting as she had subsequently been promised the money from another source. Mrs Lyons had planned to write to Sir Charles the following day to explain.

Dr Watson remained convinced that Mrs Lyons was withholding information but, despite multiple attempts to address this by rephrasing his questions, he was unable to discover anything more until he and Holmes visited together the following day. As part of his background enquiries and observations, Holmes had discovered that Mrs Lyons and Mr Stapleton frequently met and corresponded. He hypothesised that this was more than a casual social interaction and, in an attempt to encourage Mrs Lyons to divulge more, provided her with evidence of Mr Stapleton's marriage to Beryl Stapleton. This angered Mrs Lyons and led her to volunteer that Mr Stapleton had promised her (Mrs Lyons) marriage should she divorce her husband.

In his interview with Mrs Lyons, Mr Holmes diverged from best practice by asking leading tag questions. Nevertheless, the information gathered is acceptable in this instance because Mrs Lyons' replies spontaneously provided information beyond that led by the question [27]. Mrs Lyons volunteered that Mr Stapleton had dictated her letter to Sir Charles and also that Mr Stapleton had subsequently dissuaded her from keeping the appointment.

This testimony by Mrs Lyons was important for two reasons. Firstly, it provided evidence that Mr Stapleton was aware that the late Sir Charles would be waiting near to the moor gate at night on the evening of his death. Since Sir Charles was known

to avoid Dartmoor after dark, this scenario would not normally occur. Secondly, the testimony provided evidence that the unusual scenario had not arisen spontaneously, but had been deliberately engineered by Mr Stapleton. This raised serious questions about any involvement of Mr Stapleton in events later that night.

Sir Henry was chased by a large dog with phosphorescent markings shortly after leaving Mr Stapleton's house

In order to test the joint hypothesis that Mr Stapleton was responsible for the death of the late Sir Charles' Baskerville and was now planning to assassinate Sir Henry in a related manner, Holmes coordinated a sting operation. This provided a carefully managed opportunity for Mr Stapleton to make an attempt on Sir Henry's life. In brief, Sir Henry was encouraged to attend a dinner engagement alone with Mr and Miss Stapleton. After dinner, he was to walk home afterwards following a pre-agreed route across the moor. Both Mr Stapleton and Sir Henry were led to believe that Mr Holmes and Dr Watson had returned to London.

Instead of departing, Mr Holmes and Dr Watson supplemented themselves with Inspector Lestrade (Scotland Yard, London, UK) and all three surveilled Mr Stapleton's house from mid-evening onwards. As part of this surveillance, Dr Watson observed Mr Stapleton briefly visiting a locked outhouse shortly before the end of the evening. Ten or fifteen minutes later, Lestrade, Holmes and Watson all witnessed a very large black dog with phosphorescent markings and apparent flaming mouth pursuing Sir Henry along the moor path from the direction of Mr Stapleton's house. The dog was subsequently identified as a bloodhound-mastiff cross. It was shot and killed by Mr Holmes.

Mr Stapleton likely drowned in a valley mire on Dartmoor

In order to determine the location of Mr Stapleton – now presumed fugitive – Holmes, Dr Watson and Inspector Lestrade searched the house shared by Mr and Miss Stapleton. Mr Stapleton was not found, although there was considerable evidence of physical restraint and violence towards Miss Stapleton.

Following Miss Stapleton's description of Mr Stapleton's planned refuge, the three gentlemen decided to search the abandoned mine buildings at the centre of the adjacent mire. Such a search was not possible immediately due to fog, darkness and a poorly marked trail of firm grass tufts through deep and muddy water.

The following morning, Holmes, Dr Watson and Inspector Lestrade succeeded in reaching the former mine. Although they found considerable evidence that the area had been used to house a dog, they did not find any recent traces of Mr Stapleton. Nevertheless, they discovered Sir Henry's missing boot to one side of the safe route through the mire and Holmes hypothesised that it had been used to provide the dog with its target scent the night before. Based on this scenario, the presence of the boot provided evidence that Mr Stapleton had begun to cross the mire in the dark after releasing the phosphorescent dog after Sir Henry.

Since there was no evidence that Mr Stapleton had reached the centre of the mire in the dark, Holmes, Dr Watson and Inspector Lestrade concluded that Mr Stapleton had mistaken the safe path in the dark and fog and subsequently drowned. It is unclear whether they considered any alternative hypotheses, including the theoretical possibility that Mr Stapleton had retraced his steps and fled in an alternative direction after discarding the incriminating boot.

Conclusion

In this Adventure, Sherlock Holmes was engaged by Dr Mortimer on behalf of Sir Henry Baskerville to advise on the best course of action for Sir Henry, who had recently inherited the baronetcy from his uncle, the late Sir Charles Baskerville. Sir Charles' death, although considered natural by the coroner, possessed sufficient puzzling details to concern Dr Mortimer and Dr Mortimer feared the involvement of a legendary satanic hound.

Having interviewed both Sir Henry and Dr Mortimer, Sherlock Holmes linked the loss of an old boot of Sir Henry's but return of a new one to the likely involvement of an earthly, not supernatural, dog. Both an anonymous warning note received by Sir Henry and the likely involvement of a large earthly dog gave credence to a genuine future threat against Sir Henry which Mr Holmes took seriously. The presence of female perfume and use of the leader column from the *Times* newspaper indicated the involvement of an educated woman within Sir Charles' social circle.

Desk-based research, including correspondence with remote agents, provided evidence that Mr and Miss Stapleton, neighbours of the baronet, were living under an assumed name and relationship. Contrary to their presentation, they were in fact Mr and Mrs Jack Baskerville, wanted in South America for misappropriation of public funds. Mr Stapleton was therefore heir presumptive to the Baskerville baronetcy.

The discovery of the postscript of burned letter to Sir Charles and subsequent interviews by both Dr Watson and Mr Holmes led to witness testimony from Mrs Laura Lyons. Mrs Lyons stated that Mr Stapleton had promised her marriage if she were to divorce her estranged husband. Mrs Lyons additionally stated that Mr Stapleton had encouraged her to arrange and subsequently not attend a late-night appointment with Sir Charles Baskerville on the night of his death.

On the basis of this evidence, Mr Holmes coordinated a sting operation against Mr Stapleton using Sir Henry Baskerville as bait. Shortly after leaving Mr Stapleton's house to apparently walk home alone across Dartmoor, Sir Henry was chased by a large dog with phosphorescent markings. The hound was shot

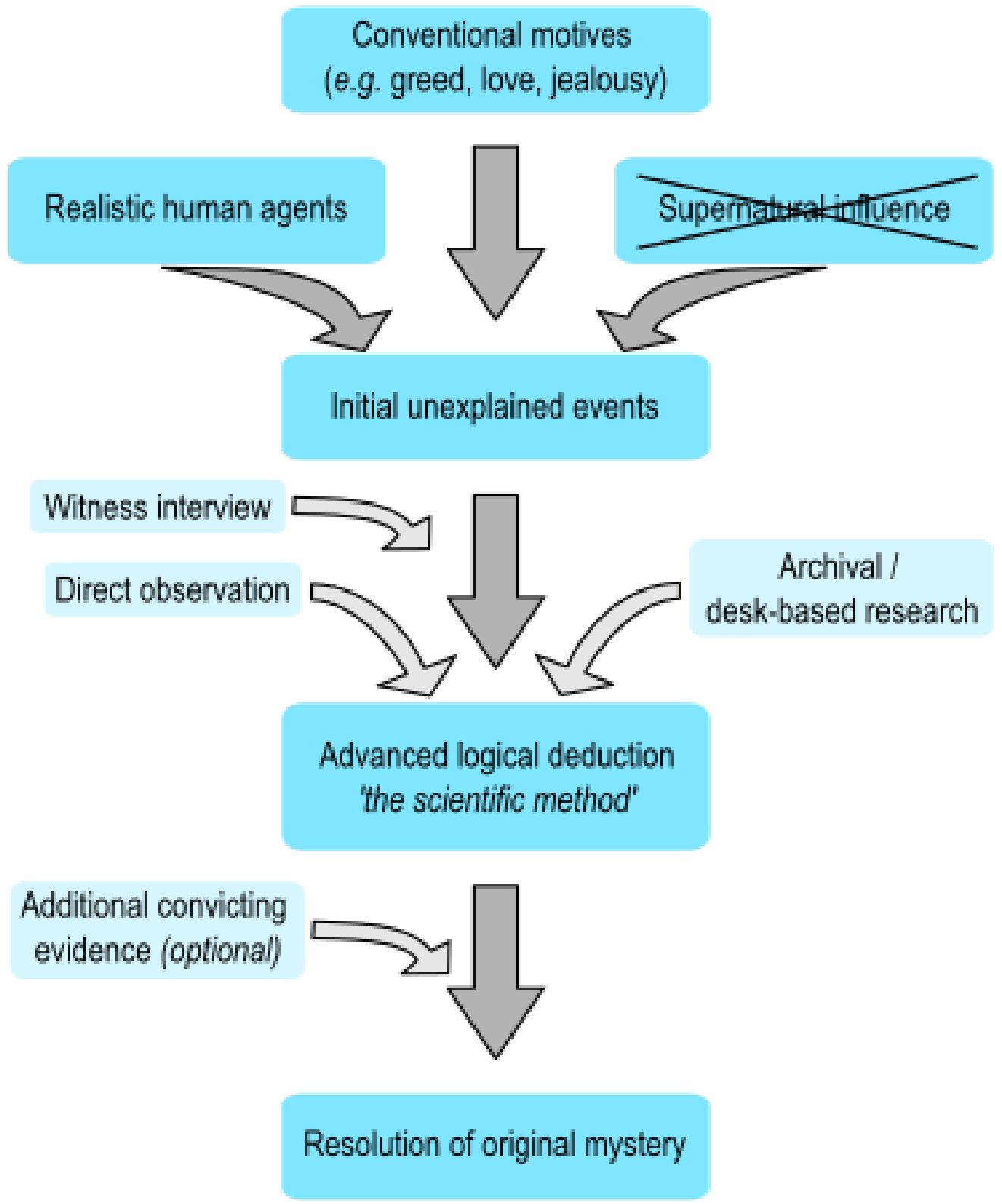

Figure 3: Model of the structure and process used in this Adventure. Conventional motives and realistic human agents give rise to unexplained events. Holmes' scientific method is applied to evidence collected during the Adventure, which enables the mystery to be resolved.

dead at the scene and circumstantial evidence indicated that Mr Stapleton drowned in Grimpen mire while fleeing.

Key to this Adventure is the juxtaposition of a dramatic gothic landscape (Dartmoor), a legendary and apparently supernatural agent (a large flaming hound), and what Mr Holmes terms 'the scientific method' within the emerging genre of detective fiction. All events in the Adventure can ultimately be explained through the real-world actions of human agents with conventional (and societally recognised) motives (Figure 3). This aligns with the accepted rational and empirical basis of scientific reasoning. Nevertheless, the juxtaposition of rational actions with the gothic and supernatural in a full-length novel advances detective fiction because the Adventure achieves dominance of the scientific method while engaging respectfully with each genre of writing and without resorting to parody. Detective fiction provides an opportunity for the author to illustrate of the power of the scientific method to explain a multitude of relatable scenarios. We expect future works to exploit this with success.

Methods

Interview technique (London)

Interviews were carried out using a mixture of open and closed questions. Specific prompts for open questions include '*This article, you say, contains all the public facts? […] Then let me have the private ones.*' [to Dr Mortimer] and '*Has anything else of interest happened to you since you have been in London?*' [to Sir Henry Baskerville].

Background enquiries

Background enquiries about the Baskerville family and local residents were made using publicly available records (including the professional registers of schoolmasters), and telegraphic and / or postal correspondence with remote agents. All information was obtained legally, following strict ethical guidelines, and stored securely at 221B Baker St, London, UK or on the person of Mr Sherlock Holmes. Any identifiable personal information was only used for the purposes of law enforcement and no personal information – other than that publicly available – is presented here.

No ethical information is available about the South African mining activities giving rise to the fortune of the late Sir Charles Baskerville.

Collection of evidence on Dartmoor

Observations and informal interviews were carried out by both Dr Watson and Mr Holmes in the Dartmoor area. Dr Watson visited Baskerville Hall openly as a visitor and companion of Sir Henry. Testimony collected by Dr Watson was in the form of social interaction with the household of Baskerville Hall and surrounding residents, and visits to the fictional local settlements of Grimpen and Coombe Tracey. Testimony was recorded within 48 hrs of collection, in the form of one or both of a private diary entry and written reports sent to Mr Holmes via London.

Sherlock Holmes visited Devon covertly. He stayed in Coombe Tracey, around 2 h drive from Baskerville Hall, and also camped on Dartmoor in what is described as a prehistoric hut. His presence in Devon was unknown to Dr Watson, Sir Henry or Mr Stapleton until the night of the convict's death. The logistical arrangements of Holmes' stay on Dartmoor (food, drink, clean linen) and agentic activities (mail collection, telegrams) were supported by a child (14 yrs; over the age of compulsory education [30]). This child – Master Cartwright – travelled from London, and was likely employed indirectly through a verbal contract with his London employer. No information is available on Master Cartwright's working hours, on child protection or on ethical approval for involvement of a minor.

Evidence collected by Holmes in Devon was in the form of direct observation of the local residents, in the immediate vicinity of Baskerville Hall and also in Coombe Tracey. This was supplemented with written reports from Dr Watson and other correspondents (see background enquiries above).

Trap of criminal activity

The sting operation arranged by Holmes used a ratio of three active gentlemen (Mr Holmes, Dr Watson and Inspector Lestrade (Scotland Yard, London, UK) to one suspect and one

dog. Inspector Lestrade was selected for his considerable expertise in law enforcement. He carried an unsigned warrant for arrest and his presence provided legitimacy for the operation. All three gentlemen were armed with handguns. No information is available on the firearms training of any participants, the maintenance record of weapons involved or of a written risk assessment or operational plan. Potential weather conditions were not included in an informal risk assessment. There is no evidence of prior ethical approval for use of a naïve civilian agent as bait in a firearms homicide trap.

Conflicts of interest

There are no conflicts to declare.

Data availability

The latest version of this article is available on Zenodo at http://doi.org/10.5281/zenodo.20630470 under a CC-BY licence.

Acknowledgements

CD would like to acknowledge the technical expertise of Chloe Ellam (St Paul's Girls' School, London) in discussion of both the individual works and wider themes of Victorian literature. CD would also like to thank Tim Sharpe (University of Basel, Switzerland) for his observation c.2005 that scientific writing does not need to conceal key information for dramatic purposes. She thanks students past and present for multiple discussions about writing style, in particular Mekhala Hanumanarasaiah (University of Bath) for suggesting a suitable internationally recognisable fictional character for the current article.

References

1. Disraeli, B. (1845). *Sybil*, Henry Colburn, London, UK.
2. Dickens, C. (1854). *Hard Times*, Bradbury & Evans, London, UK.
3. Gaskell, E. (1855). *North and South*, Chapman & Hall, London, UK.
4. Eliot, G. (1871-1872). *Middlemarch*, William Blackwood & Sons, Edinburgh, UK.
5. Gaskell, E. (1864-68). *Wives and Daughters*, Smith, Elder & Co, London, UK.
6. Dickens, C. (1843). *A Christmas carol*, Chapman & Hall, London, UK.
7. Radcliffe, A. (1794). *The Mysteries of Udolpho*, G. G. and J. Robinson, London, UK.
8. Bronte, E. (1847). *Wuthering Heights*, Thomas Cautley Newby, London, UK.
9. Shelley, M. (1818). *Frankenstein*, Lackington, Hughes, Harding, Mavor & Jones, London, UK.
10. Eliot, G. (1859). *The Lifted Veil*, Blackwood & Sons, Edinburgh, UK.
11. Verne, J. (1871). *Journey to the Centre of the Earth*, Griffith & Farran, London, UK.
12. Stevenson, R. L. (1886). *Strange case of Dr Jekyll and Mr Hyde*, Longman, Greens & Co, London, UK.
13. Darwin, C. (1859). *On the Origin of Species by Natural Selection*, John Murray, London, UK.
14. Dickens, C. (1853). *Bleak House*, Bradbury & Evans, London, UK.
15. Felix, C. (1862-3). *The Notting Hill Mystery*, Bradbury & Evans, London, UK.
16. Gaboriau, E. (1866). *L'Affaire Lerouge*, Édouard Dentu, Paris, France.
17. Collins, W. (1868). *The Moonstone*, Tinsley Brothers, London, UK.
18. Poe, E. A. (1841). *The Murders in the Rue Morgue*, Graham's Magazine, Philadelpia, USA.
19. Conan Doyle, A. (1902). *The Hound of the Baskervilles*, George Newnes Ltd, London, UK.
20. Conan Doyle, A. (1888). *A Study in Scarlet*, Ward, Lock & Co, London, UK.
21. Conan Doyle, A. (1890). *The Sign of Four*, Spencer Blackett, London, UK.
22. Dartmoor National Park Authority [Online]. Available from https://www.dartmoor.gov.uk [accessed 25th February 2026].
23. Dartmoor Preservation Association [Online]. Available from https://dartmoorpreservation.co.uk [accessed 25th February 2026].
24. Dartmoor Prison Museum [Online]. Available from https://www.dartmoor-prison.co.uk/history_of_dartmoor_prison.php [accessed 25th February 2026].
25. Baskerville, H. (1742). *Letter to my sons Rodger and John.* Private collection of the Baskerville family.
26. Devon County Chronicle. (1889). *Devon County Chronicle*, 14th May 1889.
27. Ministry of Justice (UK) & National Police Chiefs' Council (UK). (2022). Achieving best evidence in criminal proceedings: guidance on interviewing victims and witnesses, and guidance on using special measures. Smith, K. & Shaw, G. Available from https://www.gov.uk/government/publications/achieving-best-evidence-in-criminal-proceedings.
28. Bank of England inflation calculator [Online]. Available from https://www.bankofengland.co.uk/monetary-policy/inflation/inflation-calculator [accessed 26th February 2026].
29. *A fictional catalogue of British butterflies and moths.* (1885). British Museum, London, UK.
30. *Elementary Education Act 1880* (43 & 44 Vict. c.23). An Act to make further provision as to Byelaws respecting the attendance of Children at School under the Elementary Education Acts. (1880). London: HMSO.
