# Supplementary material for: Scientific writing is not a murder mystery
Source: eLife. 2026 Aug 19;15:e112853. doi: 10.7554/eLife.112853 (PMC13489623; doi:10.7554/eLife.112853)
Supplement: Supplementary file 3 [file elife-112853-supp3.pdf]

Supporting information for Dodson (2026) *Scientific writing is not a murder mystery*. eLife 2026;15:e112853 https://doi.org/10.7554/eLife.112853

Supplementary file 3: List of seventy-five perfumes considered by Sherlock Holmes

Acacia Blossom
Ambergris
Ambre
Au Fil de l'Eau
Bergamot Royale
Benzoin
Bois de Rose
Bouquet de la Reine
Bouquet Impérial
Camellia Essence
Civec
Eau de Cologne Impériale
4711 Eau de Cologne
Eau de Fleurs de Lis
Eau de Lubin
Eau de Capucine
Eau de Coquelicot
Eau de Mandorle
English Pimpernel
English Primrose
Evening Bouquet
Fleur de Grenade
Fleurs de Bulgarie
Fleurs de Provence
Fleurs d'Exposition
Fleurs de Printemps
Fleurs du Jardin
Florentine Rose
Fougère Royale
Fougère Cologne
Gardenia Water
Hammam Bouquet
Hasu-No-Hana
Heliotrope Blanc
Jasmine Royale
Jean Marie Farina
Jessamine (white)
Jicky
Jockey club
Jonquil
L'Essence de Fleurs
Lavender Water
Lemon Blossom Water
Lilac Water
Lily of the Valley
Magnolia

Malmaison
Mimosa
Mille Fleurs
Muguet
Musk
Narcisse Blanc
Neroli Royale
New Mown Hay
Orange Blossom
Orange Flower Water
Orris Root Water
Parfum d'Après-Midi
Parfum d'été
Patchouli
Potpourri
Quelques Fleurs
Rondeletia
Rose Water
Rose Saffron
Shipr
Sweet Pea Water
Sweet Violet Cologne
Tsvetochniy
Tubéreuse Impériale
Vetivert
Verveine
Violet Water
Violette Impériale
White Rose

This table was created by a mixture of AI (ChatGPT) and human input. The edited results of two AI prompts ('Please can I have a list of 90 perfumes used in the Victorian age before 1889. Please include dates and avoid duplication (including across languages).' and 'Please can you supplement this list with fifty additional perfumes, ensuring no duplication with those already provided.') was supplemented with 49 manually selected perfumes obtained by a web search.
